# The Quest for Functional Ingredients for Sustainable Aquaculture Feeds in Sub‐Saharan Africa

**DOI:** 10.1155/anu/9937988

**Published:** 2026-01-30

**Authors:** Arnold Ebuka Irabor, Rodrigue Yossa, Nurul Ahmad Fatan, Matthew A. G. Owen, Parisa Norouzitallab, Kartik Baruah

**Affiliations:** ^1^ Department of Applied Animal Science and Welfare, Faculty of Veterinary Medicine and Animal Sciences, Swedish University of Agricultural Sciences, 75007, Uppsala, Sweden, slu.se; ^2^ WorldFish Nigeria Office, IITA, Africa Rice Building, Ibadan, Nigeria, iita.org; ^3^ Aquatic Food Biosciences, WorldFish, Jalan Batu Maung Batu Maung, 11960, Bayan Lepas, Penang, Malaysia, worldfishcenter.org

**Keywords:** ecological footprint, fish meal, functional feeds, microalgae, microbial ingredients, resilient fish

## Abstract

The aquaculture sector plays a key role in ensuring food and nutritional security as well as fostering economic growth in sub‐Saharan Africa (SSA). However, as the sector continues to grow, its future faces economic, environmental, and sustainability challenges. At the core of these challenges are the substantial nutritional and health requirements of the farmed fishes that are cultured semi‐intensively and intensively. Nutrient‐balanced feed resources like fish meal and soybean are highly indispensable in most aquaculture production systems in SSA. However, these ingredients are limited, expensive, and are also in direct competition with farmed terrestrial animals and humans. Additionally, frequent disease outbreaks, especially due to the impact of climate change, pose viability challenges that are traditionally controlled using chemotherapeutics. But their indiscriminate usage has led to the occurrence of resistant microbes in the environment further adding to the challenges. Functional ingredients (FIs), derived from plants and microbial sources, are emerging as viable options to address both the nutritional and health issues of farmed fish. FIs contribute to fish health, growth performance, and resilience to disease and stress not only through their antioxidant, immunomodulatory, and antimicrobial properties but also by their nutritional benefits. The incorporation of these ingredients, into fish feeds can greatly reduce production costs, ecological footprint, and reliance on finite marine resources and synthetic drugs. However, FIs, with a few exceptions, have not been widely adopted in the aquafeed industry. This review aims to critically examine the reasons behind their limited adoption in the aquafeed industry, identifying key challenges and research gaps that hinder their widespread application. Additionally, it explores and evaluates the potential role of FIs in formulating cost‐effective and functional aquafeeds with a low ecological footprint. A particular focus is given to their role in enhancing aquaculture productivity in the SSA region, highlighting opportunities for sustainable growth and the need for further research to optimize their efficacy and commercial viability.

## 1. Introduction

Africa has a population of over 1.5 billion people, about 18% of the global population. It has been predicted that the number could increase to 2.4 billion by 2050 [1, 2]. Nourishing such a growing population presents a significant challenge for Africa’s food production systems [3]. Aquaculture is considered one of the major mitigators of food and nutritional security risks in Africa [4]. It provides affordable and high‐quality protein to over 200 million in countries such as Nigeria, Kenya, and Zambia, where population growth is rapid and economic growth is slow [5]. Beyond nutrition, the sector also serves as a source of livelihood for many households, especially in rural areas where job opportunities are minimal. According to Kaunda and Chimatiro [6], the aquaculture industry generates about US$3 billion yearly and employs over 12.3 million people (12.3% of the total population), with women making up 27.3% of this employment. Furthermore, it is predicted to employ 21.6 million people by 2050, an increase from 20.7 million by 2030, with approximately 2.4% of the workforce engaged in the fish food chain [7].

**Figure 1:**
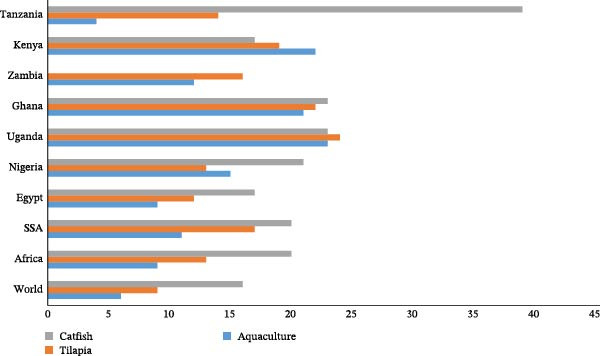
Annual growth rate in production volumes (mt) for aquaculture species, tilapia (*Oreochromis niloticus*) and catfish (*Clarias gariepinus*) during 2000–2019. Growth rates for production on the African continent, the SSA subcontinent, and the continent’s major producing nations are displayed. Notably, 70% of the aquaculture output volume in SSA is accounted for by the two leading species. Tanzania’s primary species of seaweed is being produced at a decreasing rate, but the production of smaller species like catfish and tilapia is expanding quickly. There are 51 countries in Africa and 45 in sub‐Saharan Africa. FAO FishStatj [9] provided the raw data [4].

Aquaculture was first introduced into sub‐Saharan African in the early 1920s and over the past 20 years, the sector has experienced the fastest growth rate among all the food production sectors globally [8]. In this region, several nations, such as Nigeria, Uganda, Kenya, Ghana, Namibia, and Zambia, have witnessed an increase in aquaculture production by 11% (some between 12% and 23%) annually on average since the year 2000—almost twice as fast compared with the rest of the world (Figure 1) [4]. This has helped reduce the gap between fish supply and consumer demand by over 21% annually. However, there is still a significant gap [7, 10]. Despite the surge in production, the aquaculture output in Africa, including in the sub‐Saharan Africa (SSA) region, has been relatively low (scattered between 0.18% and 0.25% annually on average) compared to the other regions, such as Asia, Latin America and the Caribbean, and Europe [4, 11, 12]. Nonetheless, given the availability of suitable land, water bodies, and adequate yet unexplored or underexplored resources, there is a good opportunity to further boost aquaculture output [13, 14]. Also to complement the common production system, such as pond culture (earthen ponds, concrete tanks, and tarpaulins), there is the possibility for the adoption of diverse production systems such as cage culture, pen culture, biofloc system, and integrated aquaculture‐agriculture systems (rice–fish culture) [5]. These systems are well‐suited to the fish species commonly cultured in the region [15, 16].

Aquaculture in SSA includes a diverse range of species, such as freshwater and marine fish, crustaceans, mollusks, and aquatic plants. Commonly farmed species are tilapia (*Oreochromis* spp.), African catfish (*Clarias* spp., *Heterobranchus* spp., and their hybrid), carp (*Cyprinus carpio*), freshwater prawn (*Macrobrachium* spp.), shrimp (*Penaeus monodon*), oysters and mussels, and aquatic plants [14, 17–19]. The culture of these species in different areas of the region is shaped by factors such as the climate, water resources (marine or freshwater), market demand, and most importantly, the availability and supply of feed and feed ingredients. The FAO report on aquaculture species cultured in the top nine producing countries in SSA between 2010 and 2021 revealed both common species of interest and the progress achieved [8] (Table 1 and Figure 2a,b).

Figure 2Trend of catfish (a) and tilapia (b) production among some aquaculture top‐producing countries in SSA from 2010 to 2021 (*Source:* Data extracted from FAO FishStatj 2023 [9]).

**Figure:**
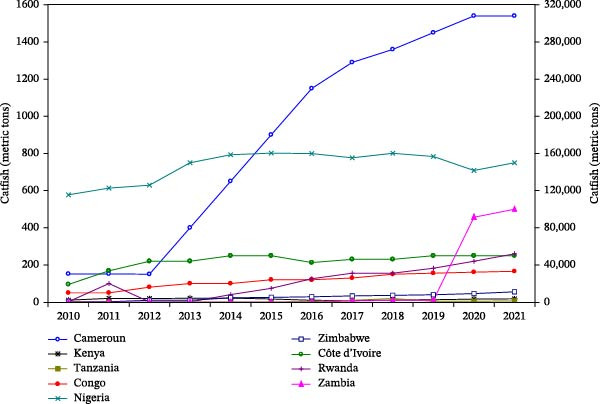
(a)

**Figure:**
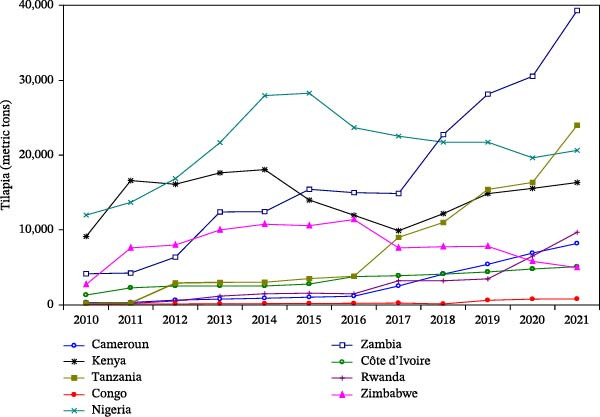
(b)

**Table 1:** The species cultured in nine countries in SSA and their yearly production indices (metric ton) from 2010 to 2021.

Year	2010	2011	2012	2013	2014	2015	2016	2017	2018	2019	2020	2021
Cameroon												
North African catfish	152	152	150	400	650	900	1150	1290	1360	1450	1540	1540
Nile tilapia	290	290	600	730	860	990	1125	2500	4080	5390	6900	8190
Congo												
Nile tilapia	55	65	95	115	130	149	173	202	90	575	739	745
North African catfish	50	50	80	100	100	120	120	130	150	156	161	166
Côte d’Ivoire												
Bagrid catfish (Brackish water)	35	68	70	70	70	70	33	50	50	50	50	50
Black catfishes (Brackish water)	0	0	0	0	0	0	0	0	0	0	0	0
Blue tilapia (Brackish water)	165	500	500	500	500	500	500	500	500	500	500	500
Blue tilapia	60	146	150	150	150	150	150	150	150	150	150	150
Nile tilapia (Brackish water)	0	100	250	250	250	250	250	220	220	220	220	220
Nile tilapia	1050	1500	1600	1600	1600	1850	2840	3000	3200	3500	3900	4200
North African catfish	60	100	150	150	180	180	180	180	180	200	200	200
Kenya												
Nile tilapia	9115	16,602	16,115	17,626	18,072	13,991	11,962	9885	12,175	14,850	15,557	16,350
North African catfish	2188	3984	3869	4230	4337	3358	1944	1606	2160	2650	3270	3450
Nigeria												
North African catfish	115,421	122,681	125,762	149,980	158,531	160,295	159,911	155,325	160,114	156,703	141,640	149,953
Tilapias (Brackish water)	0	0	0	0	0	0	0	0	0	0	0	0
Tilapias	11,989	13,675	16,872	21,681	27,987	28,284	23,706	22,534	21,734	21,743	19,652.96	20,654
Rwanda												
North African catfish	2	100	1	1.2	40	74	126	156	156	182	220	260
Nile tilapia	98	164.83	494.48	1153	1453	1533	1453	3200	3200	3450	6500	9700
Tanzania												
Nile tilapia	200	220.6	2913	2980	3000	3500	3800	9000	11,000	15,416.6	16,371.35	24,016.4
North African catfish	1	0	0	5	7	10	500	2000	3800	787.52	861.65	1264
Zambia												
Nile tilapia	4136	4234	6374	12,404	12,450	15,447	15,000	14,875.3	22,765	28,181	30,553.23	39,363.5
North African catfish	0	0	0	0	0	0	0	5.9	10	10	456.7	500
Zimbabwe												
Nile tilapia	2700	7600	8000	10,000	10,772.75	10,562.54	11,401.5	7599.24	7764.93	7818.88	5803.4	4948.94
North African catfish	2	2	10	10	23.2	25.25	28.91	32.94	36.22	39.64	45.66	55.65

*Note: Source*: Data extracted from FAO FishStatj [9].

African catfish and tilapia species represent a major fraction of the aquaculture diversity in SSA, where a combination of indigenous and introduced species is cultured to meet local demand and support economic development. Their yearly production is estimated at approximately 298,296 tons for African catfish and 60,350 metric tons for Nile tilapia [8, 14, 20]. Among the African catfish, *Clarias gariepinus*, commonly known as the African sharp‐tooth catfish, stands out as one of the most farmed species in SSA, especially in Nigeria, where it is extensively cultured and consumed [21–23]. For tilapia, Nile tilapia (*Oreochromis niloticus*) is the most cultured compared to the other species (blue tilapia and Mozambique tilapia) and this is due to seed availability, growth characteristics, and adaptability [24–26].

Beyond tilapia and African catfish, attention has increasingly turned to several other fish species with significant aquaculture potential to further strengthen the aquaculture economy in the region. This species includes *Pangasius*, *Cyprinus*, *Lates*, and *Heterotis* (for details, see Table 2) [24, 27, 29]. The introduction and culture of such species could contribute positively to overall aquaculture production in SSA. However, several challenges, such as the rising feed costs, water pollution, and most importantly, extreme weather events associated with climate change and increased incidence of disease outbreaks are hindering the future growth and sustainability of both small and large‐scale aquaculture sectors in the region [4, 13, 30–32].

**Table 2:** Potential culturable species within SSA.

Species	Market value	Availability of seed/fry	Feeding habits	Potential yield
Mullets				
* Liza lakipinds*	Good	Year‐round, but insufficient	Phylophagous/detritivorous	Extremely high yield. Does well in polyculture with tarpon, catfish, and snappers. Production: 3000 kg/ha/year
* Liza grandisquemis*				
* Mogi*, *caphalus*				
* Mugil bananensis*				
* Mogi*, *curema*				
Tarpon				
* Megalops atlanticus*	Low	Inadequate and seasonal	Predatory	Expand quickly. Because of the intramuscular bones, the flesh is of low quality. Efficient in reducing the overabundance of tilapia. Production = 4542 kg/ha/year
Exotic/native				
* Pangasius* ^∗^ *hypophthalmus* ^∗^	Good	Year‐round, but sufficient	Phylophagous/omnivorous/detritivorous	High potential for commercialization, but not fully explored
* Cyprinus carpio* ^∗^				
* Lates niloticus* ^∗^				
* Heterotis niloticus* ^∗∗^				
* Alestes* spp. ^∗∗^				
* Synodontis* spp. ^∗∗^				
Tilapias				
* Sarotharon melanotheron*	Good	Year‐round, but sufficient	Phylophagous/detritivorous	Robust and widely embraced culture. Incredibly productive yet growing slowly. Production = 4800 kg/ha/year
* Tilapia guineensis*				
Catfish				
* Chrysichthys nigrodigitatus*	Very good	Year‐round, but insufficient	Omnivorous	Practical. Grow in the culture medium very slowly. Needs artificial fields and thrives when cultured with mullets or tilapia. Production = 4542 kg/ha/year
* Bagus bayed*				
* Bagus domae*				
Snapper				
* Lutjanus goreensis*	Good	Year‐round, but insufficient	Predatory	Excellent for controlling overabundance of tilapia in ponds when grown in polyculture. 1412 kg/be/annum is the yield
* Lutjanus aegenis*				
Ten pounder				
* Elops lacerta*	Low	Inadequate and seasonal	Predatory	Rapid growth and low‐quality meat because of intramuscular bones result in a low market value. Produced 4250 kg/ha/year
Grunters				
* Pomadasys jubelini P. peroteti*	Good	Inadequate and seasonal	Predatory	Good predator in ponds with brackish water = 1412 kg/ha/year yield
* P. roperi*				
Shellfish Shrimp				
* Peanaeus notialis*	Good	Inadequate and seasonal	Detrivorous	Culture remains experimental. Culture potential is enormous
* Peanaeus monodon*				
* Macrobrachium vollenhovenii*				
Periwinkle				
* Tympanotonus fuscatus*	Very good	Inadequate and seasonal	Filter feeder	Culture potential is enormous
* Tympanotonus radula*				
Whelk				
* Thais coronata*	Low	Inadequate and seasonal	Predatory	Culture potential is enormous
* Pugillina morio*				
Bloody Cockle				
* Anadara* (*senilia*) *senilis*	Very good	Inadequate and seasonal	Planktophyagous; filter feeder	Culture potential is enormous
Crab				
* Callinectes amnicola Rochebrune*	Good	Inadequate and seasonal	Planktophyagous; filter feeder; omnivorous	Culture remains experimental
* Cardisoma armatum Herklots*				
* Paraleptuca chlorophthalmus*				
* Scylla serrata*				
* Ocypode ryderi*				
* Potamonautes perlatus*				
Oyster				
* Crassostrea gasas*	Good	Inadequate and seasonal	Detrivorous	Culture remains experimental

*Note: Source*: Kaleem and Bio Singou Sabi [24]; Ugwumba and Ugwumba [27]; Mikpon et al. [28]; Irabor et al. [29].  ^∗^ denote exotic species and  ^∗∗^ denote native species.

It is noteworthy that these challenges do not often act in isolation, but rather interact in complex ways, compounding their overall impact and creating systemic barriers to sustainable aquaculture growth. A good example of these interacting challenges is the rapid increase in the occurrence of disease outbreaks in aquaculture systems. Disease emergence in many farms is not solely the result of pathogenic microbes but is often linked to a combination of factors, which include poor feed quality that causes nutritional deficiencies, and poor environmental conditions, for instance, linked to climate change. These conditions create favorable environments for the pathogen to proliferate, weakening the defense system of the fish, making them more susceptible to the pathogens [33, 34]. Farmers have to rely on chemotherapeutics (e.g., antibiotics) and vaccination to treat or prevent such diseases, leading to further increases in operational costs. Both these approaches serve their purpose, and when used responsibly, can significantly reduce mortality. However, in many parts of SSA where the regulatory framework is limited, these chemotherapeutics are often misused or overused. These not only increase operational costs but also pose serious health risks back on the health of the final consumers of cultured fish and on the environment [35–37].

Vaccination, though effective, comes with its own limitations. It requires cold‐chain logistics, skilled personnel to administer, and proper handling. These requirements increase costs and make it difficult for small‐scale farmers to adopt. A major portion of these challenges could be avoided by feeding the farmed fish consistently and adequately with nutrient‐balanced feed. However, the ground reality is that the cost of commercial and formulated feeds remains high for many small‐scale farmers. This financial constraint pushes many to turn to alternative methods, such as experimenting with homemade or do‐it‐yourself (DIY) feeds made from locally sourced ingredients. While this approach can be budget‐friendly, it tends to lack consistency in nutrient content and may not adequately meet the dietary requirements of the fish. This deficiency can prevent the intended benefits of disease prevention and improved fish health from being fully achieved, potentially perpetuating the cycle of disease and increasing reliance on unsustainable chemotherapeutics.

To address the many sustainability challenges it faces, the aquaculture production systems in the SSA region must rely on locally available, easily accessible, and inexpensive sources of nutrients and bioactive components with broad‐spectrum health benefits. Reliance on local sources of functional ingredients (FIs) in place of unsustainable chemotherapeutics and imported, conventional feed materials could address economic, environmental, and social sustainability challenges facing the aquaculture sector. FIs are feed ingredients that not only provide nutritional value but also deliver health‐promoting benefits [38]. By including such ingredients with multifunctional benefits, aquaculture farmers in SSA can reduce reliance on traditional, high‐cost feed resources, and can achieve economic, environmental, and social sustainability goals. Importantly, FIs can be locally sourced from agro–industrial by‐products or waste streams, indigenous plants, and marine and freshwater resources, further reducing costs and supporting circular economy principles.

**Figure 3:**
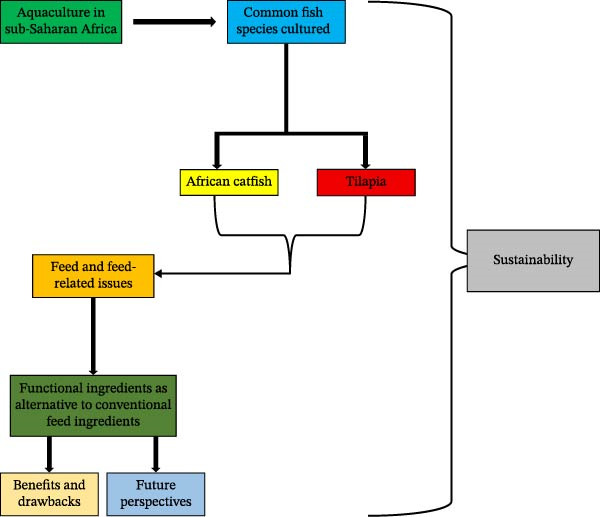
Structure of this review.

In this review, we aim to showcase the potential of FIs as innovative and sustainable alternatives to conventional feed resources in African aquaculture (Figure 3). By focusing on ingredients that deliver both nutritional value and health‐enhancing benefits, we intend to discuss how FIs can help lower production costs, decrease dependence on expensive imported feeds and feed additives, and offer practical solutions to ongoing challenges caused, for instance, by environmental changes and disease outbreaks. Special focus is given to SSA’s untapped potential, including its wealth of indigenous plants, agro–industrial waste streams, and marine and freshwater resources as viable sources of FIs. The primary goal is to demonstrate how strategies centered around FIs can pave the way for a more resilient, competitive, and environmentally responsible aquaculture industry in SSA and across the continent.

## 2. Feed and Feed Ingredients Issues in SSA

Feed and feed ingredients remain among the most critical bottlenecks hindering the sustainable growth of the aquaculture sector in SSA. Feed accounts for approximately 60%–75% of the total production cost, making it the single largest expenditure for fish farmers [24, 39, 40]. The high cost, coupled with unpredictability in the continuous accessibility of the formulated feeds and quality feed ingredients, significantly limits both the expansion and sustainability of the sector.

In response to the demand, numerous African distributors and aquafeed manufacturers have emerged in the region, directly importing branded formulated feeds. For instance, about 1,651,146 MT of feeds were imported in 2016 for African catfish and tilapia by Nigeria and Egypt, respectively [41]. However, the fluctuation in the exchange rate and rigid import procedures adversely affect these imports, contributing to the hindrance of aquaculture growth in SSA. Notably, are the scarcity and increase in price of feed ingredients and feeds resulting from the worldwide economic conflicts especially in Europe and Middle East [42]. In Ghana, the cost of imported fish feed is heavily influenced by transportation cost, taxes, and import duties, which in total accounts for about 80% of the price of feed [43]. Similarly, Nigeria relies on imported aquafeeds for about 95% of its supply, with over 70% of feed expenses attributed to these same factors [44]. It was reported that over 84% of fish farmers in Benin Republic rely solely on imported ingredients and feeds for their production [45]. In Egypt, Abdel‐Hay et al. [46] reported that feed accounts for approximately 85% of total fish production costs, largely due to high price of imported ingredients. Also, the over reliance on imported feeds due to limited domestic feed production, lack of strong investment incentives, and minimal government support was reported as major challenges of the aquaculture sector in Uganda [47].

This feed mill related drawback cut across other parts of SSA, hindering the establishment and sustainability of large‐scale feed mills. For instance, Nigeria has the highest number of fish feed mills in SSA, with mainly small‐scale producers. Unfortunately, an hourly production capacity of between 0.5 and 3 tons, accounting for around 60% of the fish feed produced locally [48]. Annually, over 47,750 metric tons of fish feed are produced in Nigeria, but meet only 12% of the total demand [49]. This significant shortfall forces fish farmers to rely on imports. Zambia also experiences the shortfall in fish feed despite having eight established feed mills producing over 30,000 metric tons annually. Which is less than the expected yearly production capacity of over 105,000 metric tons, due to the high cost of importing ingredients such as fish meal [50].

Some feed millers in the SSA region have been formulating feeds using a mix of a few locally available ingredients and imported key ingredients, such as fish meal, fish oil, maize, soyabean, groundnut, and wheat [45, 51]. However, this reliance on imports causes significant economic and environmental challenges. Fish meal and fish oil, regarded as staple components in aquafeed, are not only expensive but also pose sustainability concerns, as their production depends on overexploited wild fish stocks, leading to ecological imbalances in marine ecosystems (Table 3) [52, 53]. At the same time, climate change and global competition for crops such as maize, soybean, wheat, and groundnut have further constrained the availability and affordability of these ingredients across the region. The dependence on these imported ingredients exposes local producers to volatile international market prices and high transportation costs, inflating production expenses. These scenarios make it difficult for aquaculture farmers, particularly those run by small‐ and medium‐scale farmers, to remain profitable and competitive. This dependency also heightens the sector’s vulnerability to global supply chain disruptions, making long‐term growth and sustainability uncertain.

**Table 3:** Status report on the primary small pelagic (clupeids) populations in some SSA countries as of 2021.

Stock	Status	Recommendation
Bonga fish (*Ethmalosa fimbriata*) (Congo, Gabon, and Democratic Republic of the Congo)	Overexploited	Reduction of effort and catch down below previous harvested levels is necessary to provide an opportunity for stock repopulation that can guarantee sustainability
Sardinellas (*Sardinella aurita*, *S. maderensis*) (Côte d’Ivoire, Congo, Ghana, Togo, Liberia, Guinea Bissau, Guinea, Sierra Leone, and Benin)	Overexploited	Reduce fishing activity and harvest in all of the regions immediately and significantly (a 50% reduction is necessary)
Sardine (*Sardina pilchardus*)	Nearly overexploited	Due to the fluctuation in the species population in response to hydro climatic changes, prudence and a catch restriction under careful observation are necessary

*Note: Source*: Thiao and Bunting [52].

To address the surge in the demand for commercial fish feed, fish farmers and aquafeed companies are resorting to a DIY approach, becoming on‐farm local feed producers, developing feed using locally available ingredients [4, 45, 47]. This strategy is driven by the goal of reducing production costs and improving local accessibility for fish farmers. For instance, several feed industries in Nigeria, Kenya, Malawi, Zambia, Ghana, Cameroon, and Uganda are now producing branded local fish feed [4, 54]. However, the local feed production sector remains unviable due to the production of substandard fish feed, for instance, rough pellets, an imbalanced nutrient profile, and poor digestibility. These deficiencies stem from a mix of structural and operational constraints like unreliable supply of electricity, lack of modern processing and packaging technologies, deficient transport and storage facilities, outdated formulation methods, non‐species‐specific formulations, and use of poor‐quality ingredients to cut costs [55].

In Tanzania, for instance, imported commercial fish feeds have reached prices exceeding $1.51/kg of feed, causing a further increase in production cost and a decrease in profit margin [56]. Local feed mills are few and limited in output, with only about 10 mills producing about 323 tons per year. This has led fish farmers to produce their own farm‐made feeds (about 400–1000 kg per day), often with little scientific backing [56]. In Uganda, the production capacity of the 12 established mills is insufficient to meet the feed demand. These mills have reported a maximum weekly production capacity of approximately 5 tons since 2019 [52]. Consequently, the persistent challenges include high feed costs and compromised feed quality. Additionally, poor quality feed developed using locally available ingredients, many of which are not well studied for their nutritional properties, has led to the poor performance and poor health of the farmed animals. The effects of climate change such as fluctuating water temperatures and poor water quality have also led to increased susceptibility of cultured fish to disease outbreaks [57]. Owing to this, farmers often resort to the use of imported chemotherapeutic agents and antibiotics to manage disease incidences. However, the indiscriminate and frequent application of these substances has raised significant environmental and public health concerns, including the development of antimicrobial resistance, contamination of aquatic ecosystems, and accumulation of chemical residues in fish products [35, 57].

To address the challenges posed by poor‐quality feeds, limited feed ingredients, and frequent disease outbreaks, fish farmers in the SSA region have adopted numerous innovative and adaptive production methods and feeding strategies. One common approach involves the use of readily available domestic byproducts, such as viscera, feathers, and blood meal sourced from abattoirs. These ingredients are processed and incorporated into farm‐made feeds as cost‐effective protein sources to replace or supplement expensive fish meal and imported feeds [55, 56]. However, these practices are predominantly carried out by small‐scale fish farmers, who operate under resource‐limited conditions and rely heavily on locally available feed ingredients and traditional processing methods [58]. While such innovations demonstrate the adaptability and resilience of local farmers, they have not yet been upscaled to meet commercial aquaculture demands. The feeds produced often vary in quality and nutrient composition due to inconsistent ingredient sourcing, lack of standardized processing, and limited access to technical expertise or feed formulation technologies [55, 58]. It is, however, important to mention that the low level of industrialization and limited investment in feed manufacturing companies across SSA limit the transition from semi‐intensive operations to commercially competitive aquafeed systems [52]. Consequently, most smallholder farmers continue to depend on elementary feed production techniques that are insufficient to support large‐scale operations or ensure optimal fish growth and health. Strengthening research‐extension linkages, promoting public–private partnerships, and introducing affordable feed technologies could, therefore, play a pivotal role in bridging the gap between small‐scale innovation and commercial feed production, thereby enhancing the sustainability and productivity of the aquaculture sector in the region.

Alongside these efforts, there is increasing interest in integrating locally available FIs, such as *Moringa oleifera*, garlic (*Allium sativum*), and ginger, into fish feed formulations [24, 39, 45, 59–63]. SSA is particularly rich in such functional resources due to its vast biodiversity and abundance of indigenous plants and agro–industrial by‐products with demonstrated bioactive and nutritional potential [57, 64]. These natural FIs complement the nutritional value of unconventional protein sources like blood meal and feather meal and also enhance fish immunity, disease resistance, and growth performance through their antimicrobial, antioxidant, and immunostimulant properties [65, 66]. However, despite the region’s rich resource base, evidence remains fragmented across each FI and its usage in farming systems, with wide variability in nutritional and phytogenic profiles, optimal inclusion levels, ecological footprint, and cost‐effectiveness. As a result, there remains a need for more systematic and revisit studies to identify, characterize, and optimize the use of these FIs in aquafeeds. Such a study could play a key role in promoting sustainable, health‐enhancing, and cost‐effective aquaculture practices across SSA.

In the following section 3, we revisit and synthesize the available literature to provide an overview of FIs from plant and microbial sources, outline best‐practice inclusion strategies alongside conventional ingredients, discuss the ecological and economic aspects of using FIs, and propose research and policy priorities to accelerate their responsible usage.

## 3. FIs

**Figure 4:**
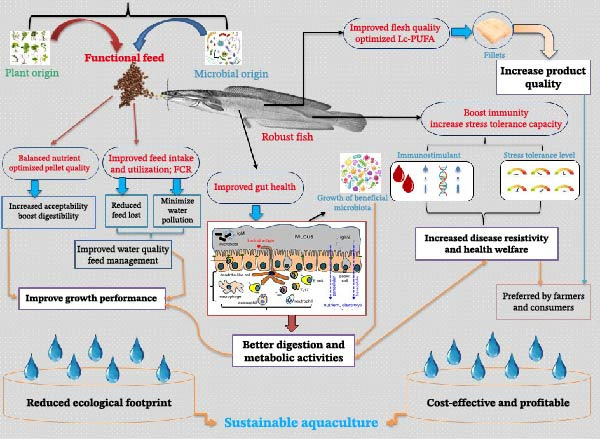
Sources and multifunctional dimensions of FIs. Feed quality‐related; fish quality‐related; general advantages (bold font).

FIs are sources of essential micronutrients, such as minerals, vitamins, and unsaturated fatty acids, as well as key macronutrients, including proteins and vital amino acids, needed for optimal fish growth and health. FIs are from different sources, such as insects, animals, plants, and microbes. However, research shows that those of plants and microbial origins are mostly explored [67–69]. These plants and their parts, that is, leaves, seeds, roots, or bark, each possess a unique composition of bioactive compounds with diverse physiological and health‐promoting properties (Table 4) [32, 71–73]. Although their protein and energy levels are lower than those of conventional fish meal, soybean, and corn meal, their incorporation in the aquafeed can supply some dietary nutrients. In addition to providing essential macronutrients, numerous studies have shown that the bioactive compounds present in the plant‐derived FIs can have a positive impact on protein synthesis, metabolic activities, and enzyme activity, thereby promoting effective nutrient digestion and absorption (Table 5). They not only enhance growth performance and overall fish health but also improve flesh composition and meat quality, which are important attributes for consumer acceptance and market competitiveness (Figure 4) [29, 32, 73, 131–137].

**Table 4:** Bioactive components of FIs and their biological functions in fish.

S/N	Bioactive components (phytochemicals)	Biological functions
1	Alkaloids	Nitrogen‐rich compounds are present in a variety of plants. Certain alkaloids, including theobromine and caffeine, can stimulate fish, altering their metabolic activities and behavior
2	Glycosides	Contains a combination of non‐sugar and sugar molecules. Certain glycosides, including cardiac glycosides, can change the heart rate and contractility of fish, which can have an impact on the cardiovascular system
3	Saponins	Glycosides with qualities akin to detergents are called saponins. They can combine with cholesterol in fish cell walls to generate compounds that promote permeability of the membrane. Additionally immuno‐modulating, saponins may improve fish immune response
4	Phenolics	Plants contain a large number of phenolic chemicals, including phenolic and flavonoid acids, known to possess antioxidant qualities. These substances can neutralize free radicals and shield cells in fish from the effects of oxidative stress
5	Organic acids	Fish use organic acids, such as malic and citric acids, in a variety of metabolic activities. They can control pH, improve the absorption of nutrients, and function as antimicrobials
6	Terpenoids	Carotenoids, steroids, and essential oils are among the many different types of chemicals known as terpenoids. Certain fish species exhibit vivid colors because of the presence of carotenoids like astaxanthin. They contain immune‐boosting qualities and function as antioxidants as well
7	Tannins	Polyphenolic substances with astringent qualities are called tannins. They can attach to proteins and create complexes that may have an impact on fish nutrition absorption and digestion

*Note:* Source: Adapted from Sepehrfar et al. [70].

**Table 5:** Selected studies on FIs as protein sources in the diets of *O. niloticus* and *C. gariepinus* in SSA.

Fish species	Functional ingredient	Dose	Effect	Reference
*Oreochromis niloticus*	*Aegle marmelos*	20 g/kg (soyabean replacement)	↑SGR, FCR, BW, and survivability	Wangkahart et al. [74]
	*Cynodon dactylon*	20% (fish meal replacement)	↑SGR, FCR, BW, and survivability	Mbokane and Moyo [32]
	*Withania somnifera* (ashwagandha or winter cherry)	20% (fish meal replacement)	↑SGR, FCR, BW, and survivability	Mbokane and Moyo [32]
	*Zingiber officinale* (ginger)	1.0% (additive)	↑SGR, FCR, BW, and survivability	Naliato et al. [75]
	*Zingiber officinale*	1.5% (additive)	↑SGR, FCR, BW, and survivability	Mahmoud et al. [76]
	*Moringa oleifera* (moringa leaf)	20% (soyabean replacement)	↑SGR, FCR, BW, PER, and survivability	Doctolero and Bartolome [77]
	*Cuminum cyminum* (cumin)	1.0% (additive)	↑SGR, FCR, BW, PER, and survivability	Deng et al. [78]
	*Manihot esculenta*	1.0% (additive)	↑SGR, FCR, BW, PER, and survivability	Aini et al. [79]
	*Tribulus terrestris*	2 g/kg (additive)	↑SGR, FCR, BW, and survivability	Attia et al. [80]
	*Astaxanthin*, *paprika*, *and capsicum*	2% (additive)	↑SGR, FCR, BW, and survivability	Amin et al. [81]
	*Piment dioica* (Allspice)	1 g/kg (additive)	↑SGR, FCR, BW, and survivability	Yilmaz et al. [82]
	*Lemna minor* (duckweed)	50% (fish meal replacement)	↑SGR, FCR, BW, and survivability	Irabor et al. [83]
	*Carica papaya* (pawpaw leaf extract)	1.98% (additive)	↑SGR, FCR, BW, and survivability	Somdare et al. [84]
	*Aloe vera*	2.0% (additive)	↑SGR, FCR, BW, and survivability	Ochingo et al. [85]
	*Cenchrus clandestinus* (kikuyu grass)	25% (fish meal replacement)	↑SGR, FCR, BW, PER, and survivability	Mbokane and Moyo [32]
	*Mitracarpus scaber*	6 g/kg (maize replacement)	↑SGR, FCR, BW, PER, FI, and survivability	Adeshina et al. [86]
	*Garcinia kola* (bitter kola)	6% (additive)	↑SGR, FCR, BW, PER, and survivability	Nyadjeu et al. [87]
	*Schizochytrium* sp.	4%, 8%, 12.5%, and 16.1% (fish meal replacement)	↑SGR, FCR, BW, and survivability	Sarker et al. [88]
	*Nannochloropsis oculata*	5% and 10% (fish meal replacement)	↑SGR, FCR, BW, and survivability	Zahran et al. [89]
	*Aspergillus oryzae*	5%, 10%, 15% and 20% (fish meal replacement)	↑SGR, FCR, BW, and survivability	Shukry et al. [90]
	*Bacillus pumilus*	0.5 g/kg (fish meal)	↑SGR, FCR, BW, and survivability	Hassaan et al. [91]
	*Bacillus amyloliquefaciens*	0.5% (additive)	↑SGR, FCR, BW, and survivability	Ridha and Azad [92]
	*Lactobacillus* sp. (dairy yogurt (DY))	0.5% (additive)	↑SGR, FCR, BW, PER, and survivability	Ridha and Azad [92]
	*Lactobacillus plantarum*	0.5% (additive)	↑SGR, FCR, BW, PER, and survivability	Mohammadi et al. [93]
	*Bacillus subtilis*	0.5% (additive)	↑SGR, FCR, BW, PER, and survivability	Mohammadi et al. [93]
	*Bifidobacterium longhum*	1.0, 2.0, 3.0, and 4.0 g/kg	↑SGR, FCR, BW, and survivability	Khalafalla et al. [94]
	*Psychrobacter maritimus*	3% (additive)	↑SGR, FCR, BW, and survivability	Makled et al. [95]
	*Saccharomyces cerevisiae* (baker’s yeast)	4 g/kg (additive)	↑SGR, FCR, BW, and survivability	Opiyo et al. [96]
	Hybrid microbial phytase (*Buttiauxella gaviniae*, *Yersinia mollaretti*, and *Hafnia* sp.)	1000FTU/kg (fish meal replacement)	↑SGR, FCR, BW, and survivability	Adeshina et al. [97]
	*Bacillus safensis* (NPUST1)	10^6^ CFU/g (additive)	↑SGR, FCR, BW, and survivability	Wu et al. [98]
	*Rhodotorula mucilaginosa*	1.0% (additive)	↑SGR, FCR, BW, and survivability	Chen et al. [99]
	Fermented poultry by‐product meal	11.17%–25.14% (fish meal replacement)	↑SGR, FCR, BW, PER, and survivability	Dawood et al. [66]
	*Sporidiobolus pararoseus*	20.0 g/kg (fish meal replacement)	↑SGR, FCR, BW, PER, FI, and survivability	Van Doan et al. [100]
	*Torulaspora* sp. *GXUS02 and Metschnikowia* sp. *GXUS03*	10^8^ CFU/g (additive)	↑SGR, FCR, BW, PER, and survivability	Liao et al. [101]
	*Hermetia Illucens* (black soldier fly larvae)	25% (fish meal replacement)	↑SGR, FCR, BW, PER, and survivability	Tippayadara et al. [102]
*Clarias gariepinus*	*Manihot esculenta* (peel)	50% (replacement for maize)	↑SGR, FCR, BW, PER, and survivability	Adewumi [103]
	*Ocimum gratisssimum* (clove basil)	2% (additive)	↑SGR, FCR, BW, PER, survivability villi length, villi width, and absorption area	Falaye et al. [104]
	*Allium sativum* (garlic)	0.5 g/kg (additive)	↑SGR, FCR, BW, PER, and survivability	Jabbi et al. [105]
	*Rosmarinus officinalis*	1.0% (additive)	↑SGR, FCR, BW, PER, and survivability	Mbokane and Moyo [32]
	*Hyphaene thebaica*	1.5% (additive)	↑SGR, FCR, BW, PER, and survivability	Khalil et al. [106]
	*Sonneratia caseolaris* (apple mangrove)	0.5 g/kg (additive)	↑WG and SGR	Aznan et al. [107]
	*Garcinia kola* (bitter kola)	1 g/kg (additive)	↑SGR, FCR, BW, PER, and survivability	Mbokane and Moyo [32]
	*Hyphaene thebaica* (*Mart*.) (Doum palm)	15 g/kg (fish meal replacement)	↑SGR, FCR, BW, PER, FI, and survivability	Al‐Khalaifah et al. [108]
	*Ipomea batatas* (sweet potato leaf)	20% (maize replacement)	↑SGR, FCR, BW, PER, FI, and survivability	Irabor et al. [109]
	*Moringa oleifera* (moringa leaf)	20% (soyabean replacement)	↑SGR, FCR, BW, PER, FI, and survivability	Irabor et al. [110]
	*Lemna minor* (duckweed)	40% (fish meal replacement)	↑SGR, FCR, BW, PER, FI, and survivability	Irabor et al. [111]
	*Zingiber officinale*	1.0%	↑SGR, FCR, BW, PER, and survivability	Ude et al. [112]
	*Carica papaya* (pawpaw seed)	20%	↑SGR, FCR, BW, and survivability	Irabor et al. [113]
	*Saccharomyces cerevisiae* (yeast)	10% (fish meal replacement)	↑SGR, FCR, BW, PER, and survivability	Dakare et al. [114]
	Autolyzed brewer’s yeast (AY)	3 g/kg (fish meal replacement)	↑SGR, FCR, BW, PER, and survivability	Adeoye et al. [115]
	*Agaricus bisporus*	5% or 10% (additive)	↑SGR, FCR, BW, PER, and survivability	Harikrishnan et al. [65]
	Microalgal strain, *Ascochloris* spp.	30% (fish meal replacement)	↑SGR, FCR, BW, PER, and survivability	Sharma et al. [116]
	Microalgae	30% (fish meal replacement)	↑SGR, FCR, BW, PER, and survivability	Agboola et al. [117]
	*Spirulina platensis* and *Chlorella vulgaris*	50% each (fish meal replacement)	↑SGR, FCR, BW, PER, and survivability	Raji et al. [118]
	*Microcystis* sp. and *Daphnia magna*	1.5, 3, 4.5 g/kg (fish meal replacement)	↑SGR, FCR, BW, PER, FI, and survivability	Tine et al. [119]
	*Spirulina platensis*	30% (fish meal replacement)	↑SGR, FCR, BW, PER, FI, and survivability	De Chavez and Bolivar [120]
	*Spirulina platensis* and *Chlorella vulgaris*	50%–70% (fish meal replacement)	↑SGR, FCR, BW, PER, FI, and survivability	Abiodun [121]
	*Spirulina platensis* and *Eisenia fetida*	50% each (fish meal replacement)	↑SGR, FCR, BW, PER, FI, and survivability	Nyangate et al. [122]
	Biofloc meal	10% and 20% (fish meal replacement)	↑SGR, FCR, BW, PER, and survivability	Ekasari et al. [123]
	*Arthrospira platensis*	50% (fish meal replacement)	↑ SGR, FCR, BW, and survivability	Rosenau et al. [124]
	*Gryllus bimaculatus* (cricket)	40% (fish meal replacement)	↑SGR, FCR, BW, PER, and survivability	Taufek et al. [125]
	*Hermetia Illucens* (black soldier fly larvae)	25% (fish meal replacement)	↑SGR, FCR, BW, PER, and survivability	Maranga et al. [126]
	*Musca domestica* (housefly maggot)	21% (fish meal replacement)	↑SGR, FCR, BW, PER, and survivability	Fawole et al. [127]
	Bacterial protein	30% (fish meal replacement)	↑SGR, FCR, BW, PER, and survivability	Adeoye et al. [128]
	*Auricularia auricula*	4% (fish meal replacement)	↑SGR, FCR, BW, PER, and survivability	Wei et al. [129]
	Defatted African palm weevil larvae	50% (fish meal replacement)	↑SGR, FCR, BW, PER, and survivability	Adeparusi et al. [130]

Abbreviations: BW, body weight; FCR, feed conversion ratio; FI, feed intake; PER, protein efficiency ratio; SGR, specific growth rate.

Microbial sources, such as *Nannochloropsis oculata*, yeast, *Isochrysis* sp., and *Schizochytrium* sp., and beneficial bacteria, offer rich potential as FIs for fish diets. These ingredients combine high nutritional components (e.g., high EPA, docosahexaenoic acid (DHA), protein, essential amino acids (methionine and lysine), lipids, and mineral content) with functional bioactive compounds (e.g., β‐glucans, mannans, and antimicrobial peptides). Such characteristics make them valuable alternatives to fish meal and fish oil, as well as unsustainable chemotherapeutics, helping to reduce Africa´s dependence on imported and often costly conventional ingredients and chemotherapeutic components that may modulate fish health, improving performance [115, 138–140]. Locally produced microbial biomass, especially on organic waste streams, could significantly lower production costs, support small‐scale aquafeed enterprises and circular bioeconomy initiatives, thereby promoting environmentally responsible and economically viable aquaculture. The growth‐ and health‐promotion effects of microbial‐based ingredients have been reported in several aquaculture species, including tilapia and African catfish, which are widely farmed in SSA. For instance, feeding African catfish a diet containing 5% dried yeast as a partial fish meal replacement resulted in an increased modulation of the intestinal microbiota [115]. Similarly, in Nile tilapia, a 4 g/kg replacement of fish meal with a combination of the yeast *Saccharomyces cerevisiae* in the diets of the fish led to significant improvement of gut health matrix [139]. Beyond those examples, numerous studies have also reported improved growth performance feed efficiency and gut health in species, such as *C. gariepinus*, *O. niloticus*, and *M. rosenbergii*, when fed with diets containing various inclusion levels of yeast species (e.g., *Wickerhamomyces anomalus*, *Kluyveromyces marxianus*, *Saccharomyces cerevisiae*, *Blastobotrys adeninivorans*, and *Cyberlindnera jadinii*) as partial replacements for fish meal and/or fish oil [141–148] (Table 5).

Numerous findings suggest that the FIs from plants and microbes have a strong potential for regional adaptation and application. They are readily available in significant quantities throughout the year in SSA. Most of the plants are extensively used as vegetables, spices, and herbs in human and livestock nutrition, making them culturally acceptable and logistically feasible as ingredients for use in fish feeds [32]. Also, the Africa’s warm climate and abundance of agricultural and agro–industrial by‐products provide favorable conditions and substrates for microbial cultivation, offering an affordable and sustainable avenue for large‐scale production of microbial ingredients. By integrating plant and microbial‐based ingredients into fish feeds, African aquaculture can improve productivity, fish welfare, and environmental sustainability while enhancing food security and livelihoods across the SSA region and beyond.

Although the potentials of FIs have been reported in numerous studies, some studies have also reported negative effects resulting from FIs, especially those of plant origin. Notably, reduced growth performance has been observed when these ingredients are used as total replacements for fish meal. For instance, *Moringa oleifera* leaf meal at over 50% replacement in *Clarias gariepinus* diets [149]. However, a partial replacement has shown substantial beneficial effects in terms of improved growth and health. Such a reduction in the inclusion levels can significantly lower feed costs and reduce dependency on imported ingredients without compromising nutritional quality. Table 5 presents the optimum inclusion levels of FIs of plant and microbial origins used as substitutes for conventional ingredients or additives recorded for the two commonly cultured species in SSA, *O. niloticus* and *C. gariepinus*.

Beyond growth promotion, these FIs also offer prophylactic and therapeutic benefits that are increasingly recognized as essential for a resilient farming system. These ingredients exhibit antibacterial, antioxidant, antifungal, anti‐inflammatory, and antiviral properties, contributing to enhanced immune function and overall physiological resilience in fish [150]. Several studies have reported the immune‐boosting potential of many of these ingredients (Table 6), which strengthen fish resilience to environmental stressors and pathogens [151, 155, 156, 175, 179]. Such properties not only reduce disease outbreaks but also minimize the reliance on synthetic antibiotics, thereby addressing environmental and public health concerns associated with antimicrobial resistance [37].

**Table 6:** Selected studies on FIs as immunostimulant in *Oreochromis niloticus* and *Clarias gariepinus* in SSA.

Fish species	Functional ingredient	Dose	Effect	Increased survival against	Reference
*Oreochromis niloticus*	*Zingiber officinale* (ginger)	1%	↑Phagocytic activity, respiratory burst, lysozyme activity, total protein, and globulin	*Vibrio vulnificul*	Naliato et al. [75]
	*Azadirachta indica* (neem leaf)	6%	↑Phagocytosis and extracellular burst activity of the blood leukocytes	*Streptococcus agalactiae*	Abdel Rahman et al. [151]
	*Thymus vulgaris* (thyme)	2%	↑Hematocrit, red blood cell, and innate immune response (myeloperoxidase activity, lysozyme activity, phagocytic activity, white blood cell, neutrophil, and monocyte counts)	*Streptococcus iniae*	Khalil et al. [152]
	*Rosmarinus officinalis* (rosemary)	0.5%	↑Hematocrit, red blood cell, and innate immune response (myeloperoxidase activity, lysozyme activity, phagocytic activity, white blood cell, neutrophil, and monocyte counts)	Aflatoxin B1	Naiel et al. [153]
	*Trigonella foenum graecum* (fenugreek)	3%	↑Histopathology, oxidative status, and immune related gene expression	*Aeromonas hydrophila*	Moustafa et al. [154]
	*Citrus sinesis* (sweet orange)	3%	↑Physiological, antioxidant, and immunostimulant activities	*S. iniae*	Mohamed et al. [155]
	*Citrus limon* (bitter lemon)	1%	↑Physiological, antioxidant, and immunostimulant activities	*Edwardsville tarda*	Mohamed et al. [155]
	*Origanum vulgare* (oregano)	2.0%	↑Haematological parameters and cytokines levels, phagocytic index, and nitric oxide level (NO) as well as serum lysozyme activity	*Aeromonas hydrophila*	Aly et al. [156]
	*Tinospora cordofolia*	5.49 g/kg	↑Antioxidative capacity, immune response, and ameliorated stress‐related markers induced by hypoxia stress	N/A	El Basuini et al. [157]
	*Ocimum sanctum*	3 g/kg	↑Immune‐antioxidant response, head kidney gene expression, and intestinal architecture	*A*. *hydrophila*	Rahman et al. [158]
	*Cuminum cyminum*	2.0%	↑Innate immune response	*Pseudomonas fluorescens*	Dey et al. [159]
	*Tribulus terrestris*	2 g/kg	↑Sex reversal, immunological, and haemato‐biochemical parameters	N/A	Ghosal et al. [160]
	*Pimento dioica* (allspice)	15 g/kg	↑Antioxidant, and immunological responses, and resistance	*Plesiomonas shigelloides*	Yilmaz [161]
	*Curcuma longa* (turmeric)	2.5%	↑Improved and maintained blood‐immune homeostasis parameters	N/A	Pereira et al. [162]
	*Aloe barbadensis* (aloe vera)	10 g/kg	↑Red blood cell, mean corpuscular volume, mean corpuscular hemoglobin, respiratory burst activity, lysozyme, and myeloperoxidase	N/A	Syed et al. [163]
	*Moringa oleifera*	5.8 g/kg	White blood cells, lysozyme, and phagocytic activities	*A. hydrophila*	El‐Son et al. [164]
	*Nauphoeta cinerea*	10%	↑↑Hematocrit, red blood cell, and innate immune response (myeloperoxidase activity, lysozyme activity, phagocytic activity, white blood cell, neutrophil, and monocyte counts)	*Aeromonas hydrophila*, *Citrobacter freundii*, *Pseudomonas* sp., and *Enterobacter amnigenus*	Tubin et al. [165]
	*Aspergillus* sp. (vinasse)	2.0 g/L	↑Histopathology, oxidative status,	Aflatoxins	Rulli et al. [166]
	*Tenebrio molitor*	10%	↑Physiological, antioxidant, and immunostimulant activities	*Pseudomonas* sp.	Tubin et al. [167]
	*Scenedesmus obliquus*	5 mg/L	↑Physiological, antioxidant, and immunostimulant activities	N/A	Silva et al. [168]
	*Lactobacillus plantarum*	10^8^ CFU/g LP	↑Haematological parameters and cytokines levels, phagocytic index, and nitric oxide level (NO) as well as serum lysozyme activity	*S. agalactiae*	Van Doan et al. [100]
	*Hermetia Illucens* (black soldier fly larvae)	10%, 20%, 40%, 60%, 80%, and 100%	↑Immune response and ameliorated stress‐related markers induced by hypoxia stress	N/A	Tippayadara et al. [102]
	*Chrysomya putoria* larvae	50%	↑Immune‐antioxidant response, head kidney gene expression, and intestinal architecture	N/A	Agbohessou et al. [169]
*Clarias gariepinus*	*Artemisia afra*	12%	↑White blood cells, lysozyme, and phagocytic activities	*A. hydrophila*	Mbokane and Moyo [170]
	*Moringa oleifera*	10 g/kg	↑White blood cells, lysozyme, and phagocytic activities	*A. hydrophila*	Ekelemu et al. [171]
	*Aloe barbadensis* (*aloe vera*)	1.0%	↑Haematobiochemical parameters (red blood cells, hematocrits, and hemoglobin. Platelets, white blood cells, lymphocytes, monocytes, and granulocytes) and kidney histology	N/A	Adegbesan et al. [172]
	*Ocimum gratissimum* (African basil)	12 g/kg	↑Haematobiochemical parameters (red blood cells, hematocrits, and hemoglobin. Platelets, white blood cells, lymphocytes, monocytes, and granulocytes)	*Listeria monocytogenes*	Abdel‐Tawwab et al. [173]
	*Vernonia amygdalina* (bitter leaf)	10 mg/kg	↑Haematobiochemical parameters (red blood cells, hematocrits, and hemoglobin. Platelets, white blood cells, lymphocytes, monocytes, and granulocytes)	N/A	Alagoa and Osakwe [174]
	*Zingiber officinale* (ginger)	20 g/kg	↑Haematobiochemical parameters (red blood cells, hematocrits, and hemoglobin. Platelets, white blood cells, lymphocytes, monocytes, and granulocytes)	N/A	Purbomartono et al. [175]
	*Allium sativum* (garlic)	5 g/kg	↑Haematobiochemical parameters (red blood cells, hematocrits, and hemoglobin. Platelets, white blood cells, lymphocytes, monocytes, and granulocytes)	*Pseudomonas aeruginosa*	Tiamiyu et al. [176]
	*Carica papaya* (pawpaw seed)	20 g/kg	↑Haematobiochemical parameters (red blood cells, hematocrits, and hemoglobin. Platelets, white blood cells, lymphocytes, monocytes, and granulocytes)	N/A	Ekokotu et al. [177]
	*Azadirachta indica* (neem leaf)	7%	↑Phagocytosis and extracellular burst activity of the blood leukocytes	*Micrococci* spp., *Bacillus subtilis*, *Escherichia coli*, and *Pseudomonas fluorescens*	Ubiogoro et al. [178]
	*Bacillus subtilis*	1 × 10^10^ CFU/kg	↑↑Hematocrit, red blood cell, and innate immune response (myeloperoxidase activity, lysozyme activity, phagocytic activity, white blood cell, neutrophil, and monocyte counts)	*Aeromonas hydrophila*	Hamed et al. [179]
	*Musca domestica*	21%	↑Histopathology, oxidative status,	N/A	Fawole et al. [180]
	*Hermetia Illucens* (black soldier fly larvae)	172 g/kg (75% fish meal replacement)	↑Haematobiochemical parameters (red blood cells, hematocrits, and hemoglobin. Platelets, white blood cells, lymphocytes, monocytes, and granulocytes) and kidney histology	N/A	Fawole et al. [181]
	Biofloc meal	10% and 20% (fish meal replacement)	↑Haematobiochemical parameters (red blood cells, hematocrits, and hemoglobin. Platelets, white blood cells, lymphocytes, monocytes, and granulocytes)	N/A	Ekasari et al. [123]
	Defatted African palm weevil larvae	10 mg/kg	↑Haematobiochemical parameters (red blood cells, hematocrits, and hemoglobin. Platelets, white blood cells, lymphocytes, monocytes, and granulocytes)	N/A	Adeparusi et al. [130]
	*Gryllus bimaculatus* (cricket)	40%	↑Haematobiochemical parameters (red blood cells, hematocrits, and hemoglobin. Platelets, white blood cells, lymphocytes, monocytes, and granulocytes)	N/A	Taufek et al. [125]
	Earthworm meal	2.5%	↑Haematobiochemical parameters (red blood cells, hematocrits, and hemoglobin. Platelets, white blood cells, lymphocytes, monocytes, and granulocytes)	*Bacillus* spp. and *Lactococcus raffinolactis*	Nugraha et al. [182]
	Yellow mealworm	45%	↑Haematobiochemical parameters (red blood cells, hematocrits, and hemoglobin. Platelets, white blood cells, lymphocytes, monocytes, and granulocytes)	N/A	Sankian et al. [183]
	*Spirulina platensis*	40 g/kg	↑Phagocytosis and extracellular burst activity of the blood leukocytes	*Aeromonas hydrophila*	Purbomartono et al. [184]
	*Chlorella vulgaris*	50 CL	↑Phagocytosis and extracellular burst activity of the blood leukocytes	N/A	Raji et al. [118]
	*Spirulina* and*β-glucan*	1.5 mg/L	↑Phagocytosis and extracellular burst activity of the blood leukocytes, histopathology, and oxidative status	Chlorpyrifos	Mokhbatly et al. [185]
	*Arthrospira platensis*	3%, 5%, and 7%	↑Phagocytosis and extracellular burst activity of the blood leukocytes, histopathology, and oxidative status	*Aeromonas hydrophila*	Nasir et al. [186]

Importantly, several of these ingredients, such as those of plant origin, also influence reproductive performance, improving gonadal development, hormone regulation, and gamete quality in key aquaculture species, including *C. gariepinus* and *O. niloticus* [187–189]. The reproductive performance and organs of *C. gariepinus* have shown improvement when certain FIs are included in their diets. For example, dietary inclusion of 200 mg/kg ginseng extract in the diets of *C. gariepinus* accelerated sexual development, a substantial rise in sperm quality, improved spermatozoa ultrastructure, gonadal–somatic index, and increased serum follicle‐stimulating hormone levels [135]. Additionally, *Croton zambesicus* (rushfoil) and *Sesamum indicum* (sesame) seed powder at 100 mg/kg were found to enhance the egg production capability of female *C. gariepinus* [187]. While dietary supplementation of *Garcinia kola* at 100 g/kg enhanced sperm viability and quality in males [190]. Inclusion of *Desmodium adscendens* leaf meal at low levels (0.015 g in females and 0.02 g in males) resulted in improved gonadal‐somatic indices, ovary size, and milt quality [188]. In *O. niloticus*, 2% inclusion of *Allium sativum* (garlic) improved sperm and egg quality [191], and supplementation with *Tribulus terrestris* extract (750 mg/kg) significantly enhanced reproductive performance [192].

Although the effectiveness of these plant‐based FIs on fish fertility improvement has been established; however, the molecular mechanism behind this remains insufficiently known. Most bioactive substances found in these ingredients have been reported to boost fish fertility by neutralizing free radicals, promoting detoxification, and lowering oxidative stress in fish [70]. However, it is important to note that certain androgenic components in these ingredients also interfere with reproduction and reduce the spawning rate by altering the sex gametes. For instance, phytoandrogens; dehydroepiandrosterone, androstenedione, and testosterone have been reported to modify the reproductive gametes and performance of certain fish species [189]. These components have, in some instances, been effectively applied to control prolific breeding in Nile tilapia and other related species. Additionally, the phytochemicals such as tannins, steroids, alkaloids, flavonoids, and saponins, have been found to inhibit the actions of enzymes necessary for the synthesis of estrogen and testosterone [189], providing potential for targeted control of reproduction. A wide array of these ingredients, including *M. oleifera*, *Mangifera indica* (mango), *Azadirachta indica* (neem leaves extract), *Gossypium herbaceum* (cotton), *Psidium guajava* (guava), *C. papaya* (pawpaw), *T. foenum graecum*, *Pinus* spp. (pine trees), and *A. vera* have been tested for such functions with promising results [193, 194].

Furthermore, research has shown that these plant‐based FIs confer antioxidative protection against stress‐induced lipid peroxidation, a common consequence of intensive aquaculture and environmental stressors [195–202]. Notably, Ismail et al. [203] reported high resistance to microplastic‐induced stress in *O. niloticus* fed diets with *Amphora coffeaeformis* additive at 1%–7.5% dietary inclusion levels. Also, *O. niloticus* fed diets with 2 mL/kg of *Panax ginseng* extract as a supplement showed high resistance against stress caused by exposure to 1/5 of lethal atrazine [204]. The increased resistance to chlorpyrifos by *C. gariepinus* was reported to be influenced by the dietary inclusion of *C. papaya* in the feeds at 0.25 L/kg of body weight [205].

Collectively, these findings underscore the marked potential of FIs to sustain aquaculture in SSA through offering a holistic approach to improving fish health and productivity. However, to fully harness their potentials, there is a pressing need for comprehensive mechanistic, nutritional, and long‐term field studies that define optimal inclusion levels, evaluate species‐specific responses, and explore synergistic interactions among different FIs. Strengthening research and innovation in this area will provide the scientific foundation necessary to support the large‐scale adoption of FIs as core components of sustainable aquaculture feed systems across the SSA region.

It is noteworthy to mention that the well‐studied FIs are not limited to plant and microbial origins; some are also of animal and insect origins. The animal‐ and insect‐based FIs are currently being investigated by various research groups for their potential applications, ranging from growth promotion to immunostimulation [125, 130, 181–183]. The animal‐based FIs include blood meal and feather meal, while insect‐based ones include black soldier fly larval, African palm weevil larvae, cricket, mealworm, and earthworm meals. Yet, from both ecological footprint and cost‐benefit aspects, plant‐ and microbial‐derived ingredients have, however, been recommended in the literatures. Future translational research is needed to upscale the local production of FIs from microbial sources using regionally available resources, such as agro–industrial and household waste streams. This includes optimizing fermentation and bioprocessing technologies suitable for small‐ and medium‐scale enterprises.

## 4. Life Cycle Assessment (LCA) and Cost–Benefit Analysis (CBA) of FIs

Assessing the ecological footprint and cost implications of functional feed production using FIs is essential for ensuring the sustainability of aquaculture systems in SSA. LCA and CBA are the most effective tools to evaluate the carbon footprint and cost implications of feed production [206, 207].

### 4.1. LCA

**Figure 5:**
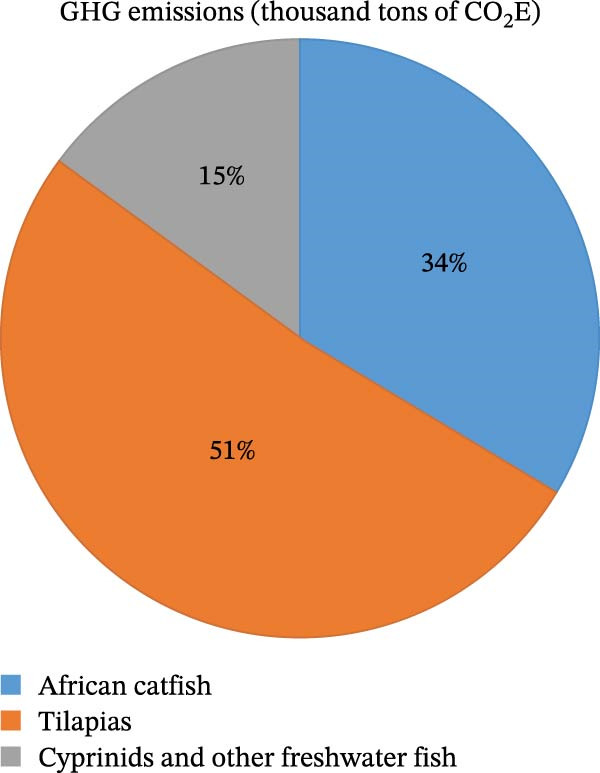
GHG emissions from the production of some common species in SSA between 1970 and 2020 (extracted from Kaleem and Bio Singou Sabi [24], Jang and Yamazaki [209], and MacLeod et al. [210]).

LCA provide insights into resource utilization, for example, for novel ingredients/feed production and quantifies impacts, such as greenhouse gas emissions, eutrophication, loss of biodiversity, and other adverse environmental effects [208]. Some studies have compared the environmental impacts of feeds formulated using FIs (either as a partial or total replacement for conventional ingredients) with those of traditional fish meal or fish oil–based diets (Figure 5). These FIs include micro‐ and macroalgae, blood meal, feather meal, insect meal, and agricultural byproducts [15, 211–213]. For instance, McKuin et al. [214] evaluated the LCA of various FIs–based feeds against conventional fish‐based feed using ecological indices, such as environmental toxicity, global warming, and eutrophication. Their results showed that the ecological footprint of fish‐based feeds was higher by over 50%. Furthermore, it was established that among the FIs–based feed, blood meal–based feed had a relatively more negative impact on the environment. In another study, Bartek et al. [215] compared the amount of DHA emitted from using two different types of feed ingredients, namely, fish meal and algae. It was found that algal‐based feeds generated lower emissions per tonnage of DHA produced. In fact, over 20 MW h of lower energy in the form of heat was needed for algal‐based feed when compared with that of the fish‐based feed. Furthermore, the fish‐based feed had a greater impact on water use and terrestrial ozone generation, exceeding five and 20 times, respectively, compared to that of algae‐based. Similarly, Deprá et al. [216] evaluated the environmental impact of different algae species, including *C. cohnii* cultivated using glucose as a carbon source, to determine indicators of acidification, eutrophication, global warming, and land use. Their results revealed that fish‐based feeds had a high environmental impact (178 ton CO_2_ eq, 0.9 ton SO_2_ eq, 0.3 ton PO_4_ eq, and 900 m^2^ land per ton of DHA). In a similar study, Bartek et al. [215] reported lower values (60 ton CO_2_ eq, 3.6 ton SO_2_ eq, 0.02 ton PO_4_eq, and 3000 m^2^ of land per ton of DHA) for fish‐based diets. However, the values were still considerably higher than those of algal‐based feeds. This discrepancy in the values among the studies could be attributed to the variation in the methodology.

When comparing cultivation and extraction methods, McKuin et al. [208] demonstrated that the microalgae cultivation stage has the greatest overall environmental impact in the LCA of amino acid extraction from defatted *Nannochloropsis* and fish meal. The key ecological hot spots were the use of urea and pure liquid carbon dioxide; and the manufacturing processes that included was the solvent‐based oil extraction methods. Although the processes involved in defatting *Nannochloropsis* to extract amino acids had considerably lower ecological impacts than that of fish meal from a small pelagic fish bio‐refinery. Factors like land use, biotic resources, global warming potential, water use, and eutrophication were identified as major concerns for marine microalgae systems. In another comparative study on the LCA of various non‐fish‐based and fish‐based feeds, Ghamkhar and Hicks [211] found that fish‐based feeds exerted stronger negative ecological impacts compared to those of plant and microbial origins. Further evaluation showed that plant‐based ingredients contributed the least ecological burdens. Hence, formulating feeds without fish‐based ingredients (fish meal and fish oil) will help to reduce drastically the reliance on biotic resources and improve environmental sustainability. Similarly, Maiolo et al. [217] reported that the energy demand per unit of product for fish‐based feed was high resulting in severe ecological impacts. However, insect‐based feeds required far less energy, even after accounting for energy and human labor inputs during the production processes of insect‐based feed.

Although fish‐based feeds are associated with higher environmental footprints, it is noteworthy that the global fishery sector accounts for only 3% of the total carbon footprint, despite representing approximately 15% of the world’s fish biomass [218, 219]). A major part of these emissions arises from harvesting and freezing processes. However, even with a relatively small carbon footprint, fisheries still exert pressure on biotic resources and ecosystems [220]. For example, nearly 5 million tons of fish are harvested annually, but the overall impact on biodiversity remains modest when compared to agricultural practices, even with the inclusion of massive fishing methods, such as trawling, considering purse seine fishing techniques are employed [221].

Using locally sourced FIs can further reduce the carbon footprints in aquafeed production. By minimizing transportation and GHG emissions, locally produced plant‐ and microbial‐based ingredients can markedly contribute to enhancing both economic and environmental sustainability [222, 223]. Additionally, consumers are also increasingly favoring environmentally friendly products, which can enable farmers ensure profitability and sustained livelihood. Moreover, locally sourced ingredients tend to be fresher and better controlled in quality [224, 225]. This reduces the risk of spoilage and contamination that occur during long‐distance transportation, resulting in higher‐quality feed, healthier fish, lower mortality rates, and greater overall production efficiency. Ultimately, localizing FIs production is both environmentally and economically advantageous, contributing to a more sustainable future for aquaculture.

### 4.2. CBA

Considering the economic advantages of FIs, numerous studies have shown that they can significantly improve growth performance and feed efficiency in a range of aquaculture species [226–230]. These improvements resulted in higher yields and lower feed costs per unit of biomass produced [207], providing a clear economic advantage. A meta‐analysis by Glencross et al. [231] revealed that replacing fish meal with plant‐ or microbial‐proteins reduced feed costs by 15%–30% when inclusion levels remained below 40%. Larsen et al. [232] reported a 15% cut down on production cost using microbial‐based protein source in place of fish meal, translating to a saving of about US$0.30 per kg of fish produced. In Saudi Arabia, feed devoid of animal‐based ingredients used in the production of *O. niloticus* recorded optimum growth performance and survivability, consequently a cut down in production cost by about US$2.03 per kg [233]. In that study, the cost to produce 1 kg of fish dropped from US$3.13 in the control diet to US$2.02 with 20% replacement. Similarly, *Pangasius* fed DIY formulated diets using agricultural byproducts (palm kernel nut, rice bran, and tufo dregs) performed optimally compared to those fed control diet. This impacted positively on the production cost with a reduction of over 50% (from US$0.62 to US$0.30) [234]. A similar outcome was observed when replacing 85% of fish meal with fermented copra meal, which reduced production cost by US$0.25 per kg [235]. However, higher replacement levels (above 50%) have been associated with reduced growth performance and feed conversion efficiency, potentially offsetting cost benefits.

In some SSA countries, such as Rwanda, Uganda, Kenya, and Tanzania, large‐scale replacement of fish meal with plant‐ or microbial‐based ingredients remains limited. However, several studies highlight the cost implications of relying solely on fish meal. In 2023, fish meal prices have risen from about US$850 to US$1287 per mt [47], while in East African countries, the cost rose from US$800 to about US$1350 per mt within a year. Similar trends were observed in Bangladesh, where fish meal price increased by 15% within a year reaching $983.64 per ton [236]. Adoption of FIs has already shown promising results elsewhere. For instance, large‐scale aquafeed production using microbial‐based protein (100,000 tons annually) demonstrated high potential to replace fish meal and soyabean meal. Less than US$1000 was spent to produce over a ton which was far more cost‐effective compared to fish meal and soybean meal‐based feeds [237]. Also, the use of microbes recorded some level of success in the cost of feed production, scalability, and acceptability. A significant improvement in growth performance and health status was recorded in Nile tilapia and African catfish when fed diets with dietary inclusion of microbial ingredients in a study by Anany et al. [238] and Ogbuagu et al. [239]. This confirms the potential of microbial‐based ingredients in feed cost reduction without compromising the well‐being of the cultured fish. The plant‐based feed market is also expanding, with projections for the European market showing an increase from 3.6% in 2023 to over 13.4% in 2033 [240]. In Norway, a contrary finding was reported as the production costs of microalgae‐derived feeds was relatively high, estimated at US$17.49 per kg of dry matter. It was still below the cost of fish‐based feeds. For cost effectiveness, an optimal production cost of about US$4.37 per kg of dry matter has been proposed. Incorporating decomposed feather meal as a replacement for fish meal in fish feed resulted in over 75% cut down on feed production cost; from US$1.10 to US$0.30 per kg [241]. On a large‐scale basis, microbial‐ and plant‐based fish feeds were estimated to cost around US$800 per metric ton, compared to US$1800 for fish meal–based feeds Jones et al. [242]. Furthermore, microbial‐based FIs were more cost‐effective due to optimum digestibility which results in initial growth rate.

The cost benefits of FIs of plant and microbial origins have been looked at from fish growth performance, feed conversion ratio, and feed production. However, the benefit is further amplified by their health‐related benefits. Extracts derived from microbial‐ and plant‐based ingredients have been shown to improve immune responses and gut health in fish [137, 243]. In *O. niloticus* production, Hossain et al. [137] recorded a significant reduction in the chemotherapeutic costs from US$2.21 to US$2.04 per kg. Similarly, a Brazilian fish farmers reported a US$60.0 reduction in chemotherapeutic expenses and US$0.56 less per kg in feed costs when using FIs for feed formulation [137]. These findings imply how FIs can lessen dependence on synthetic antibiotics, improves food safety, and mitigate antibiotic resistance while lowering the cost of disease management. Since disease control and mortality rate has a direct impact on farm profitability, these benefits strengthen the economic rationale for the quest of novel FIs and their adoption in aquafeed formulation. By improving feed efficiency and increase resilience, these feeds align with consumer demand for healthier and antibiotic‐free seafood products. This potentially opens up new market opportunities, allowing producers to command premium prices for their products [244].

It is, however, important to highlight that not all reports documented the cost‐effectiveness of FIs. For instance, a study carried out on *O. niloticus* using a certain microbial additive, such as the yeast *S. cerevisiae* as synbiotics or probiotics was found to be less cost‐effective, primarily due to the high purchase cost of the yeast and complex processing requirements [245]. Nonetheless, the long‐term advantages such as increased growth, increased feed efficiency, and decreased disease‐related losses might outweigh these initial costs. Furthermore, economies of scale and improvements in production technologies are likely to drive down costs as the adoption rate grows, opening for a wider range of feed and feed additive producers.

**Figure 6:**
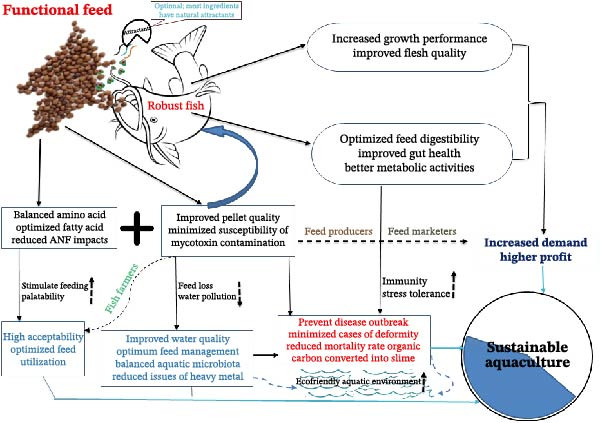
Potentials of functional feed (shown with black arrow and text in bold font). The resultant impact on fish and environment; benefit to fish producers and marketers; health‐related effects.

In addition to the earlier mentioned advantages, local production minimizes long‐distance shipping and transportation costs by eliminating fuel costs, logistics, handling, and potential tariffs or import duties associated with importing materials from other regions or countries [246]. The use of locally sourced FIs helps to simplify the supply chain, leading to a more predictable and stable supply. This also minimizes the risk of supply disruptions encountered in international shipments, thereby ensuring consistent production schedules and lower costs associated with delays or sudden shortages [247, 248]. It also supports local economies by creating a positive feedback loop, as fish farmers and suppliers can improve operations and reduce prices. This economic boost also leads to the development of better infrastructure and further cost reductions [249]. In addition, exploring these locally sourced FIs encourages the production of more tailored feed to meet the specific nutritional and health needs of cultured fish species [250–252] (Figure 6). While the economic benefits of these ingredients are increasingly evident, a drawback lies in scaling up production to meet industrial demand in the SSA region and beyond. Achieving consistent and large‐scale output will require investment in infrastructure, technological innovation, and coordinated research–industry–farmer partnerships to ensure sustainable and cost‐effective feed production.

## 5. Limitations in the Use of Locally Sourced FIs as Alternative Feed Ingredients

Plants and microbes with functional potentials have been identified as promising and sustainable alternatives to conventional feed ingredients in aquaculture. However, some limitations, especially those associated with their effects on the growth performance and nutrient utilization, have been reported. For instance, a study had shown that if microbial protein (e.g., yeast and fungal proteins) in the diet exceeds 30% inclusion level, it can negatively affect the growth of the fish [253]. In addition, various studies have reported that these feeds may lack certain essential nutrients required for optimal fish health and performance [136, 254–257]. While plant‐based FIs contain proteins, carbohydrates, vitamins, and minerals, but they are deficient in some of the nutrients such as vitamin C and E required by fish [137, 254]. Furthermore, a major point of discussion in aquaculture nutrition research is the appropriate level of replacement of fish meal with plant or microbial sources. Many researchers highlight the presence of antinutritional factors (ANFs), including alkaloids, phenol, tannin, saponin, and lignin, as a critical constraint [258–262]. The concentration of these components varies with plant type and age and can impede the active digestion process and the utilization of the available nutrients in the feed [261, 262]. Furthermore, the issue of high levels of protease inhibitors in some plant‐based FIs which include glucosinolates, allergens, gossypol, phytoestrogens, tannins, saponins, antivitamins, lectins, and phytoestrogens can also hinder fish growth and nutrient availability [258, 259].

Although the different parts of plant such as the leaf, bark, root, seed, flower, and stem have been considered as FIs, but the leaves are the most utilized till date [263, 264]. However, high‐fiber content in leaf meals pose the issue of palatability, acceptability, and digestibility, thereby adversely affecting the growth of the fish. The use of feed attractants can help improve the palatability of these plant‐based FIs [265], although some of these ingredients exhibit better palatability and are naturally more acceptable to fish thaqn others [266]. Besides the growth performance, the health status (like gut morphology and microbiota composition) of fish can also be adversely affected by the ANFs contained in the leaves. For instance, *C. gariepinus* fed diets with over 50% dietary inclusion level of moringa leaf meal as a replacement for fish meal exhibited alterations in the liver and intestine tissue structure [149]. Supplementing feeds with exogenous enzymes such as xylanase, β‐glucanase, β‐mannanase, and proteases can mitigate these effects by improving digestibility and nutrient availability [267]. However, this increases feed production costs. The necessity and cost‐effectiveness of enzyme inclusion depend on the type and proportion of ingredients replaced [137, 268–272].

Feed digestibility is further influenced by ingredient composition, processing methods, and digestive physiology of the fish. Poor digestibility can not only reduce feed efficiency but also contribute to nutrient waste and water quality deterioration. Amer et al. [273] reported low digestibility levels in *O. niloticus* fed diets with over 60% dietary inclusion level of protein extracted from moringa as a replacement for fish meal. The ANFs contained in plants such as moringa, neem, and *aloe vera*, have been shown to increase with an increase in the dietary inclusion level of the ingredients [255], further hindering the digestibility of the formulated feed. However, heat treatments during grinding, extrusion, and enzyme supplementation can degrade many ANFs, thereby improving digestibility [274, 275].

Another limitation involves the complexity of feed formulation using plant‐ or microbe‐based FIs [256]. Ensuring nutritional adequacy requires specialized knowledge of nutrient composition, processing techniques, and species‐specific dietary requirements [276]. This poses a challenge for small‐scale feed producers in developing regions, often resulting in nutritionally imbalanced feeds that impair fish growth and health [137]. Establishing standardized formulation protocols and monitoring systems for key cultured species is, therefore, critical. It is, therefore, necessary to make standard protocols for feed formulations for various cultured species and monitoring policies to ensure these standards are adhered to.

Diets produced using locally sourced FIs could reduce pressure on wild stocks (e.g., clupeids in the SSA region) and minimize the GHG emissions associated with animal‐based diets. However, overexploitation of these alternative ingredients could also introduce new environmental risks. The over‐dependence on certain plant‐based FIs may create unhealthy competition since they serve as herbs to humans or encourage unsustainable agricultural practices, such as the excess use of fertilizers and pesticides, which contribute to environmental pollution, soil degradation, and water resource depletion. Finally, research on the ecological footprint and CBA of aquafeeds formulated with combined FIs remains limited. Broader and more integrated LCA that include indicators, such as biodiversity loss and energy use, are needed to better examine sustainability [277, 278]. Comparative studies between fish meal–based and FI–based diets should also assess impacts on fish physiology, gut microbiota, and host–microbe interactions, which remain poorly understood [208]. Furthermore, large‐scale production of FI–based feeds may still depend on fossil energy inputs, affecting their greenhouse gas emission profiles [278]. Future research should, therefore, prioritize comprehensive LCA and technoeconomic assessments to optimize both sustainability and scalability of locally sourced FIs.

## 6. Strategies Towards Sustainable Adoption of FIs as Alternative Feed Ingredients

The potential of the locally sourced FIs in SSA is enormous. Accounting for over 10% of the world’s total number of identified feed ingredients, points at its diversity and sustainability. Some have been studied as a potential source of proteins, while others as feed additives. Exploring them as alternatives to the high cost and scarce conventional ingredients to produce novel FF is significant towards achieving food security in SSA. Small‐scale fish farmers stand to profit, particularly as commercial diets, synthetic hormones and chemotherapeutics are expensive and difficult to get. Their inclusion in the diets of cultured fish species can therefore help reduce operational costs, especially due to importation of ingredients and limited resources. Furthermore, FIs and FFs are associated with reduced ecological footprint.

Enhanced growth, reproduction performance, and reduced disease incidence in numerous cultured fish species have been achieved with the dietary inclusion of these FIs in some countries. This has contributed to the increased desire for such to be adopted in SSA. In rural Indonesia, over 46% of the fish farmers treated and enhanced the disease resistance potential of cultured fish species using FIs derived from plant and microbial origins [279]. Furthermore, it was also recorded that the ingredients were indigenous hence readily available and accessible at a low cost. Similar practices and levels of success were reported for China, Vietnam, Bangladesh, and India in studies carried out by some researchers [280–286]. The relevance of indigenous ingredients in the profitability and sustainability of fish farming is significant. Hence, can provide cost‐effective feed ingredients for protein and immunostimulants in SSA. Formulation of FFs using FIs is not a complex affair; it requires minimal technology and resources. Therefore, feed producers and farmers only need to be informed about the potential benefits and standard protocol concerning nutrient requirements based on species and age. Such approaches will allow the effective transition from conventional ingredients to more sustainable and eco‐friendly ingredients (FIs).

## 7. Conclusion and Future Perspectives

The adoption of locally sourced FIs in fish feeds across SSA remains limited, with most research confined to laboratory or small‐scale trials. The lack of translation of experimental findings to commercial application continues to hinder progress. Nevertheless, incorporating FIs, particularly those derived from plants, microbes, and/or agro–industrial by‐products, in the aquafeed formulation represents a viable solution for addressing the challenges faced by small‐scale fish farms in the region. These challenges, as mentioned above, are the high cost of conventional feed, poor feed quality, genetical issues, and recurrent disease outbreaks. Increased efforts are needed to bridge the gap between research findings and practical application, as many fish farmers and feed producers are unaware of the potential of locally available FIs as affordable and sustainable feed ingredients. Governments, academic institutions, and research institutes should, therefore, establish educational programs for small‐scale fish farmers and feed producers to promote the use of these ingredients in fish farming. Initiatives such as farmer training, demonstration farms, and extension programs can enhance understanding of FI use, feed formulation, and handling practices.

Overtime, it has been established that high inclusion levels (above 50%) of certain FIs can negatively affect growth performance, feed utilization, and fish health. This is often linked to ANFs, imbalanced amino acid profiles, or suboptimal digestibility. Consequently, these ingredients should be applied at moderate inclusion levels to improve resilience and physiological performance without compromising growth. In conclusion, locally sourced FIs hold potential to transform aquaculture in SSA by reducing dependency on imported fish meal and fish oil, enhancing food and nutrition security, and supporting environmental sustainability. However, realizing these benefits requires a balanced approach combining technological innovation, institutional support, and effective policy frameworks to ensure that the use of FIs leads to economically viable, nutritionally adequate, and ecologically sound aquaculture systems across the African continent.

## Funding

We would like to acknowledge the Norwegian Agency for Development Cooperation (NORAD), under Grant SAF‐21/0004 managed by WorldFish. We also wish to acknowledge ResiChar project (jnr: 2023‐1338‐2) funded by the Swedish Board of Agriculture and co‐funded by the European Union.

## Conflicts of Interest

The authors declare no conflicts of interest.

## Data Availability

Data sharing is not applicable to this article as no datasets were generated or analyzed during the current study.
